# Fast, facile synthesis method for BAL-mediated PVP-bismuth nanoparticles

**DOI:** 10.1016/j.mex.2020.100894

**Published:** 2020-04-19

**Authors:** Roberto Vazquez-Munoz, M. Josefina Arellano-Jimenez, Jose L. Lopez-Ribot

**Affiliations:** a Department of Biology and South Texas Center for Emerging Infectious Diseases, The University of Texas at San Antonio, San Antonio, TX, USA; b The University of Texas at San Antonio, San Antonio, TX, USA (former); c Department of Materials Science and Engineering, The University of Texas at Dallas, Dallas, TX, USA (current)

**Keywords:** Bismuth nanoparticles, Synthesis method, Nanoantibiotics

## Abstract

Bismuth is a water-insoluble non-toxic metallic element used in a wide array of pharmaceutical products, cosmetics, and catalysts, among others. Yet, the research regarding the use of bismuth nanoparticles (BiNPs) for antimicrobial treatments is scarce. Most of the current protocols for synthesizing BiNPs suitable for medical uses cannot be easily replicated in non-specialized laboratories. The objective of this work is to provide a fast, facile and economical method for synthesizing BiNPs. Bismuth nanoparticles were synthesized by a chemical reduction process, in less than 1 h, in a heated alkaline glycine solution; by the chelation and reduction of the bismuth (III) ions using dimercaptopropanol (BAL) and sodium borohydride respectively, and then coated and stabilized by polyvinylpyrrolidone (PVP). The resulting PVP-BiNPs were characterized by UV–Vis spectrophotometry and transmission electron microscopy (TEM).

• We describe a simple, rapid and inexpensive method for the synthesis of bismuth nanoparticles.

• This method allows synthesizing small nanoparticles with an aspect ratio close to one.

• Bismuth nanoparticles have antimicrobial properties, this easy-to-replicate protocol may further the research on bismuth nanoparticles for biomedical applications.

## Graphical abstract

**Figure:**
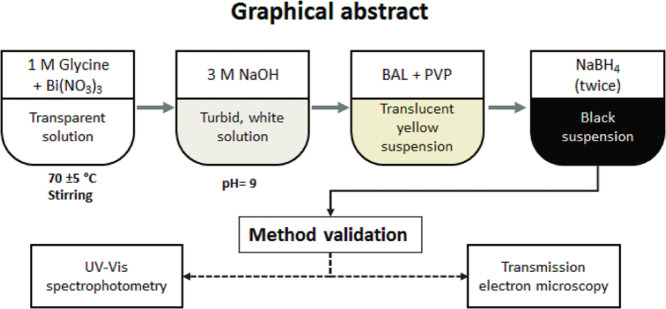

**Specifications Table**

**Table:** 

Subject Area:	*Materials Science*
More specific subject area:	*Synthesis of metallic nanoparticles*
Method name:	*Synthesis of Biocompatible BAL-mediated PVP-Bismuth nanoparticles for biomedical applications*
Name and reference of original method:	*This is an optimized method based on different methods of bismuth nanoparticles available in the literature.*
Resource availability:	*N/A*

## Background

Bismuth is a water-insoluble metallic element used in a wide array of medical applications, because it is considered non-toxic for humans (Lethal Intake >5–20 g/day/Kg, for years) [8], [14]. When bismuth is chelated with hydroxyl or sulfhydryl containing molecules, its water solubility and biocompatibility are both increased. The water solubility and lipophilicity of bismuth are substantially enhanced when bismuth ions (Bi^3+^) are complexed with small lipophilic molecules, such as dimercaptopropanol (BAL) [2]. BAL is an FDA approved drug [15] for treatments of metal poisoning [1].

Bismuth is used in the manufacture of pharmaceutical products, cosmetics, catalysts, pigments, electronics, and alloys. Water-soluble biocompatible bismuth complexes are used in health and cosmetics products and medicine. Also, bismuth and compounds present antimicrobial properties. It has been demonstrated that it exhibits high antibacterial activities against several bacterial species, including *Clostridium difficile, Helicobacter pylori, Escherichia coli, Pseudomonas aeruginosa, Proteus mirabilis*, and *Staphylococcus aureus* [6], [9]. BAL-bismuth compounds display increased antibacterial activity [20]. Yet, it has been barely studied for synthesizing antimicrobial nanoparticles (nanoantibiotics).

Bismuth–based nanostructures have been used for different applications, such as photocatalytic oxidative desulfurization processes [10], [11]. There are only a handful of studies regarding the synthesis and evaluation of bismuth nanoparticles for antimicrobial treatments. Usually, the synthesis methods for BiNPs require specialized equipment [5], [13] or controlled conditions [3], [7], [18], [19]. As such, most current protocols for synthesizing biologically suitable BiNPs cannot be replicated in non-specialized laboratories without great difficulties [2], [4], [12]. Facilitated protocols for nanomaterials synthesis expand the research for biomedical applications [16]. Here we propose a fast, facile and economical method for synthesizing BiNPs that does not require the use of advanced equipment. The justification of this research is grounded on the potential antibiotic properties of BiNPs. These nanoparticles may be potent nanoantibiotics, but their antimicrobial activity remains vastly explored.

## Method details

### Required reagents and equipment

*Reagents*: bismuth nitrate [Bi(NO_3_)_3_*5H_2_O], sodium borohydride (NaBH_4_), 2,3-dimercapto-1-propanol (BAL), sodium hydroxide (NaOH), polyvinylpyrrolidone MW=10 KD (PVP-10 K), and glycine. All reagents were purchased from Sigma Aldrich (MO).

*Equipment*: 200 ml beaker, stirring hot plate, stir bar, thermometer, pH-meter, 12 ml plastic tubes, 1000 µl pipette, 200 µl pipette, 50 mL plastic tube, aluminum foil.

### Procedure

#### Preparation

(1) The beaker, thermometer, pH-meter, and the stir bar must be perfectly clean, washed with distilled water.
(2) The following stock solutions were prepared using Milli Q water (or distilled water): 1 M glycine, 3 M NaOH, 3 mM PVP, and 1 M NaBH_4_.

NOTE 1: NaBH_4_ loses its activity very fast when diluted in water, it should be freshly prepared, immediately before using it.
NOTE 2: PVP-10 K molecular mass is 10,000. For a 3 mM solution, 0.3 g of PVP were diluted in 10 ml of Milli Q water.
NOTE 3: For a typical reaction, low volumes can be used: as an example, for a single-synthesis reaction we prepared (in Milli Q water or distilled water) the following: 1 M glycine (20 ml), 3 M NaOH (5 ml), 3 mM PVP (5 ml), and 1 M NaBH_4_ (10 ml).

(3) Bismuth nitrate and BAL are used as received. BAL has a strong odor, and it should be opened and handled inside a chemical hood. The use of gloves and masks, and all other appropriate safety measures and pertinent protective equipment, is highly encouraged during the synthesis process.

### Synthesis of the PVP-BiNPs

BAL-mediated PVP-BiNPs were synthesized by the chemical reduction of bismuth ions in an organic solution. The following is a full step-by-step description of the proposed synthesis method.

(1) 20 ml of 1 M glycine solution were heated up to 70 ± 5 °C, under continuous vigorous stirring. This temperature range must be kept through all the synthesis process.

NOTE: temperature is crucial for the proper synthesis. Lower temperatures result in a highly unstable suspension that precipitates within minutes after the synthesis.

**Fig. 1:**
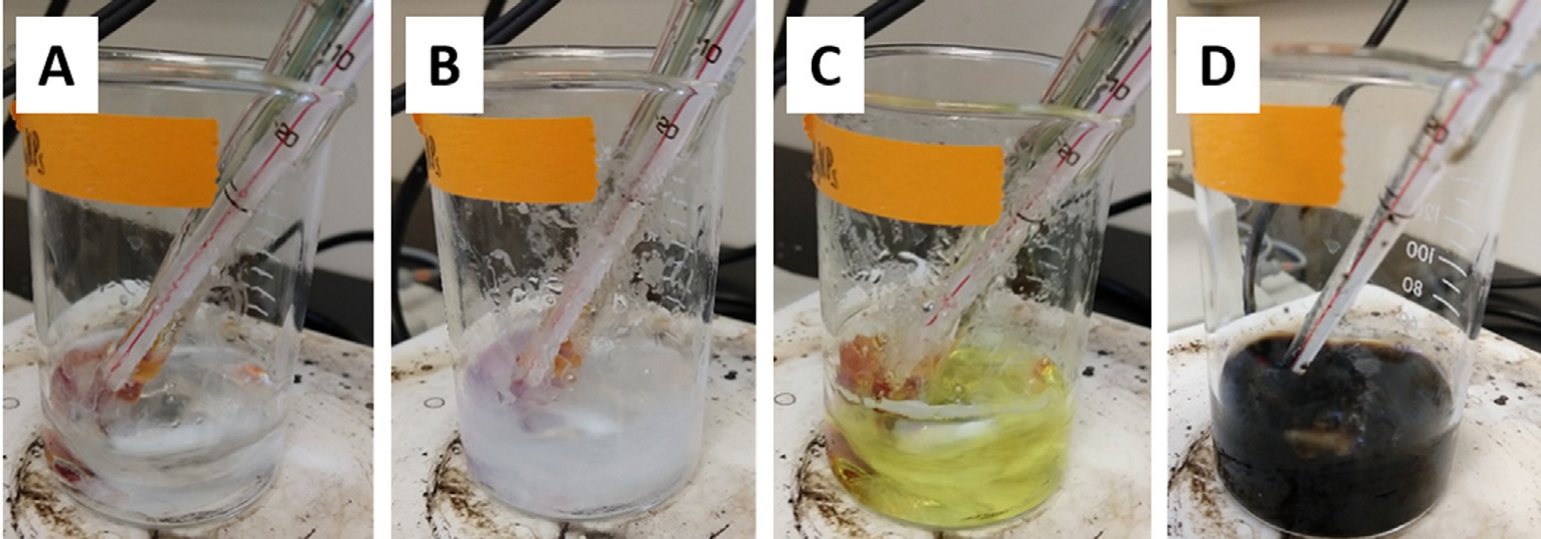
Synthesis process: from the initial colorless bismuth salt solution (A), it turned to a turbid white color after the addition of NaOH and PVP (B); when BAL was added it changed to a translucent yellow color (C). Finally, when NaBH_4_ is added, the solution immediately turned to pitch-black color (D). (For interpretation of the references to color in this figure legend, the reader is referred to the web version of this article.)

NOTE: pH is likely to drop during the synthesis process, more NaOH can be added to keep it at 9.

(2) 146.2 µg of the Bi(NO3)_3_*5 H_2_O crystals were added to the pre-warmed glycine solution (for an initial 15 mM bismuth solution).
(3) After ~2 min, enough volume of 3 M NaOH was added to raise the solution pH to 9. This turns the solution from transparent to a turbid white color Fig. 1A and B. This alkaline pH is kept during the whole synthesis process.

(4) After 3 min, 75 µL of 8.1 M of 2,3-dimercaptopropanol (BAL) were added, rapidly turning the turbid whitish appearance to a translucent bright yellow color Fig. 1C.
(5) Immediately, 3 mL of 3 mM PVP-K10 were added to the stirring suspension.
(6) Finally, about 1 min later, 5 mL of 1 M NaBH_4_ were added dropwise. The suspension rapidly turns to a deep black color Fig. 1D.

NOTE: NaBH_4_ induces an exothermic reaction increasing the temperature of the solution, it is important to add it slowly.

(7) Approximately 3 min later, another 2 ml of 1 M NaBH_4_ were added dropwise and it was left for vigorous stirring for ~10 additional minutes.

NOTE: If the protocol was followed as written, and the volume of NaOH was around 1.5–2 ml, the final concentration of the total bismuth should be around ~9.3–9.5 mM (~1254–1985 µg ml^−1^). There may be other bismuth species in the solution but those can be removed by washing the BiNPs (see below).

(8) The BAL-mediated PVP-BiNPs black suspension was stored in a Falcon® plastic tube and cooled down to room temperature and posteriorly stored at 4 °C.

### Optional: washing the PVP-BiNPs*

The Bismuth nanoparticles can be washed to remove other bismuth species. For the washing process, we performed the following procedure:

(1) BiNPs are centrifuged at 4000 rpm for 25 min and then washed with Milli-Q water, twice;
(2) BiNPs were centrifuged again, then left to dry until they form a dry powder, then kept at 4 °C, in a light-protected container;
(3) Dried BiNPs can be suspended in sterile Milli Q water.

*If BiNPs are washed, the concentration of bismuth can be adjusted as desired, by weighting the BiNPs in the desired volume of Milli Q water.

## Method validation

After the method was standardized, the BAL-mediated BiNPs were synthesized in more than 10 rounds, on different days, to verify the reproducibility of the protocol. The measurement of the BiNPs size was performed on randomly selected different rounds of synthesis, for the TEM and the DLS analysis. The statistical analysis was performed on the Prism 8 (GraphPad Software Inc) software.

### Characterization of the BAL mediated PVP-BiNPs

#### UV–Vis spectroscopy

**Fig. 2:**
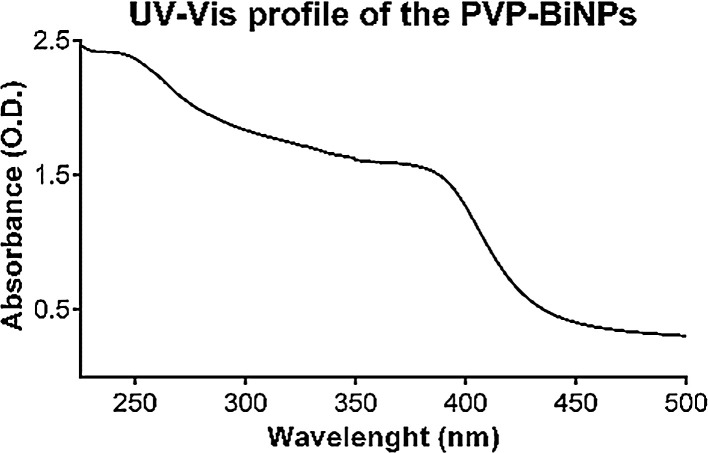
The UV Vis absorbance profile of the BAL-mediated PVP-BiNPs reveals a peak around the 400 nm position.

BAL-mediated PVP-BiNPs absorbance profile was collected in a UV–Vis-NIR Cary 500 spectrophotometer (Agilent Technologies), in a wavelength range from 225 to 500 nm, in 1 nm steps. Bismuth Nanoparticles showed a constant absorbance from 225 nm to 500 nm, then decreases at λ = 385 nm. Results from the UV–Vis spectrophotometry analysis suggest the transformation from bismuth (III) ions to bismuth nanoparticles Fig. 2. This UV–Vis profile is similar to the one reported by Wang & Kim for PVP-BiNPs [17].

#### High-resolution transmission electron microscopy

**Fig. 3:**
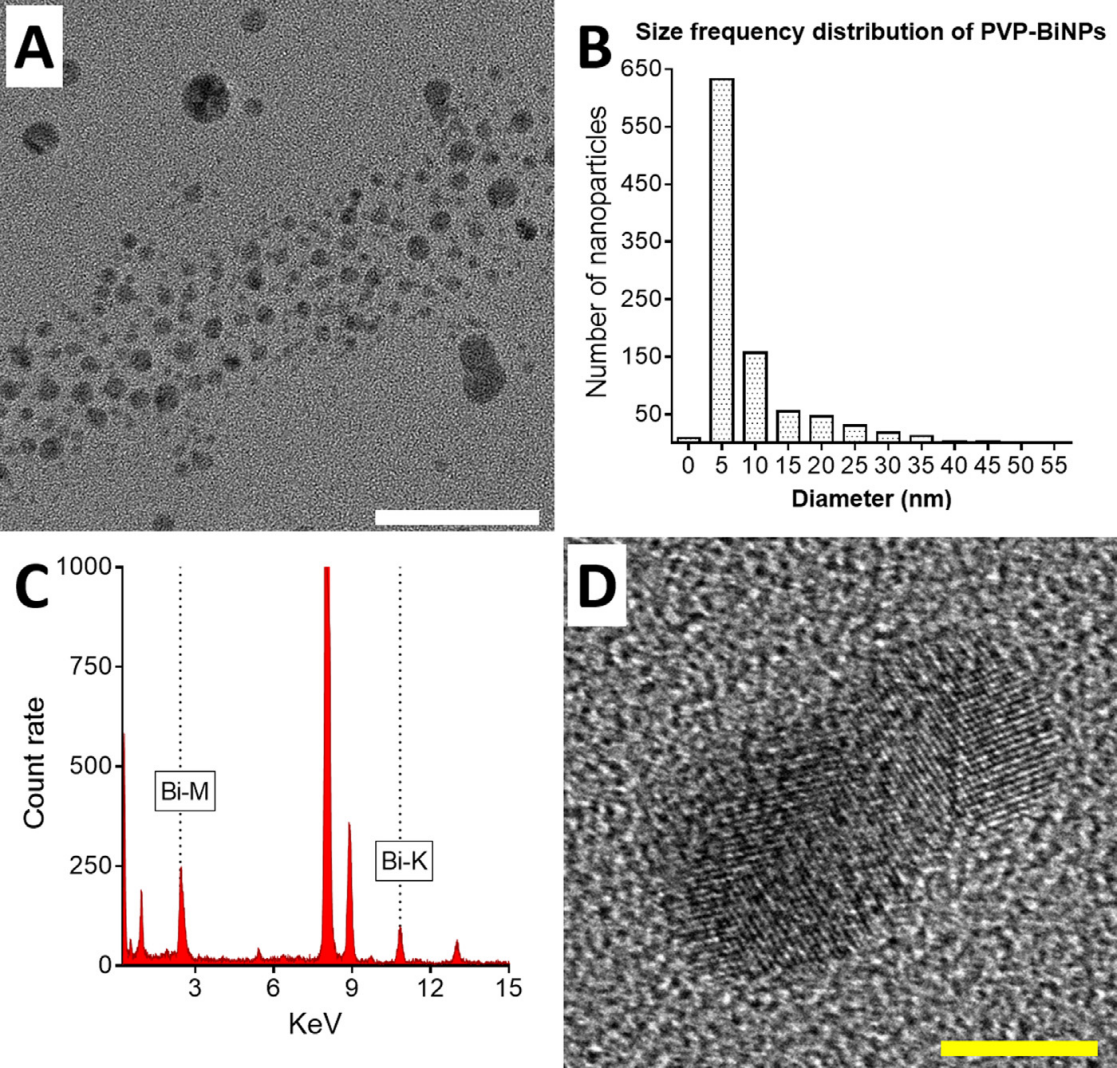
Electron microscopy characterization. HR-TEM images reveal that the BAL-mediated PVP-BiNPs were small nanoparticles, most of them with an aspect ratio close to 1 (A). The size distribution for the statistical analysis is shown in panel B. An EDS Analysis confirms the presence of Bismuth on the nanoparticles (C). A closer view reveals the crystalline arrangement of the nanoparticles. Scale bar: white =50 nm (A), yellow=5 nm (D). (For interpretation of the references to color in this figure legend, the reader is referred to the web version of this article.)

10 µL from the PVP-BiNPs suspension were deposited on Type-B Carbon-coated copper grids (Ted Pella Inc.) and left to dry overnight. The BiNPs were analyzed in a JEOL 2010-F HR-TEM (Jeol Ltd.), with an accelerating voltage of 200 kV. TEM images confirm the presence of small nanoparticles, with an aspect ratio of close to 1 Fig. 3A. The statistical analysis of the frequency distribution size (performed on Prism 8, GraphPad Software Inc.) reveals that the average diameter of the nanoparticles was 8.57 ± 7.52 nm (*n* = 964) Fig. 3B. An Energy Dispersive X-ray spectroscopy (EDS) analysis was performed to assess the chemical elemental composition of the nanoparticles. The AgNPs were deposit in a copper grid (Ted Pella) and analyzed using an EDAX collector Fig. 3C. The HR-TEM analysis of a single particle confirms the crystalline arrangement of the bismuth nanoparticles Fig. 3D.

#### Dynamic light scattering (DLS) analysis

**Fig. 4:**
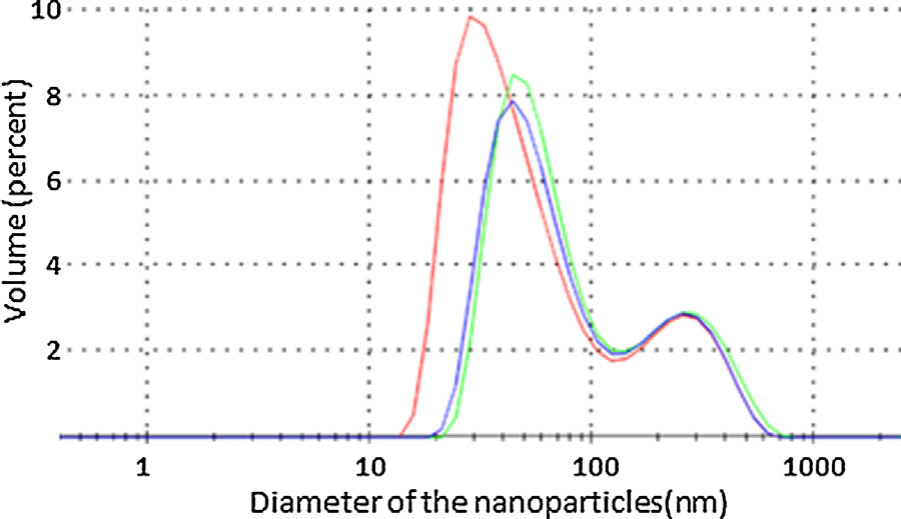
The DLS analysis reveals that the bismuth nanoparticles size is around 20 nm, although some nanoparticles and clusters are larger than 100 nm.

The Hydrodynamic size of the BAL-mediated PVP-BiNPs was determined by a DLS analysis. Briefly, the bismuth nanoparticles -diluted in Milli Q water- were transferred to a DTS1070 cell and analyzed in a Zetasizer Nano ZS (Malvern Panalytical), at room temperature, in triplicate. The BAL –mediated PVP-BiNPs hydrodynamic size is 22.5 ± 0.06 Fig. 4. The hydrodynamic size is greater than the metallic core observed on electron micrographs. This can be attributed to the extended PVP chain-like molecules from the coating, which hydrated under the aqueous environment. According to Wang et al., FTIR demonstrates that PVP interacts chemically with bismuth [17], resulting in PVP-coated bismuth nanoparticles.

## Conclusion

In summary, we describe a facile, fast, and economical method for the synthesis of BAL-mediated PVP-BiNPs, using basic laboratory instruments and reagents readily available in most laboratories. This method can be easily replicated for research or educational purposes in nanotechnology-related fields, thus encouraging the research for BiNPs as nonantibiotics. These BAL-mediated PVP-BiNPs were small spheroids (<15 nm). The potential of BiNPs as promising nanoantibiotics remains relatively unexplored, despite multiple advantages, such as low cost, low toxicity, and potent antimicrobial activity.

## Declaration of Competing Interest

The authors declare that this research study was performed in the absence of any commercial relationship that may be a potential conflict of interest. The funders had no role in study design, data collection, data analysis, decision to publish, or preparation of the manuscript.
